# Psychosocial Support for Parents, Infants, Children, and Adolescents with Variations of Sex Characteristics: Results from a Pan-European Survey

**DOI:** 10.3390/bs14090832

**Published:** 2024-09-17

**Authors:** Martin Gramc, Surya Monro, John Stephenson, Jürg Streuli

**Affiliations:** 1 Institute of Biomedical Ethics and History of Medicine, University of Zurich, 8006 Zürich, Switzerland; martin.gramc@ibme.uzh.ch (M.G.); jstreuli@dialog-ethik.ch (J.S.); 2 Centre for Gender and Africa Studies, Faculty of the Humanities, University of the Free State South Africa, Bloemfontein 9301, South Africa; 3 Department of Criminology, Sociology, and Social Policy, School of Social Sciences and Humanities, Loughborough Campus, Loughborough University UK, Loughborough LE11 3TU, UK; 4 Department of Allied Health Professions, Sport and Exercise, School of Human and Health Sciences, Huddersfield University, Queensgate, Huddersfield HD1 3DH, UK; j.stephenson@hud.ac.uk

**Keywords:** DSD, intersex, variations of sex characteristics, multidisciplinary teams, psychosocial care, paediatrics

## Abstract

Early psychosocial support for parents/legal guardians who have children with variations of sex characteristics (VSCs) is crucial in helping avoid potentially harmful medical procedures. Psychosocial support, including peer support, can help parents/legal guardians choose the best care path for their child, and it remains important throughout childhood. However, there is a lack of data on the provision of psychosocial support for families with a child who has VSCs. We sought knowledge about the timing and types of psychosocial support, and the level of implementation of psychosocial support amongst health and psychosocial care professionals and peer supporters. A survey was conducted using a purposive sample of healthcare professionals and members of peer support groups across Europe. A total of 301 responses were received and analysed using descriptive and inferential methods. The survey results showed that psychosocial support primarily addresses diagnostic procedures, medical treatment, and medical interventions. Whilst the majority of healthcare professionals aspired to have psychosocial support provided at the point where a diagnosis of VSCs was suspected, this was only reported as current practice by a minority of respondents. Overall, the survey indicates that there is a need for greater implementation of psychosocial support, and more collaboration between healthcare professionals and peer support groups in caring for children with VSCs and their families.

## 1. Introduction

Variations of sex characteristics (VSCs) are chromosomes, hormones, genitals, and reproductive organs that are considered atypical by the medical establishment [1]. These variations are also referred to as *intersex* by some activists and policy makers [2,3], or as *disorders of sex development* (*DSD*) by many medical practitioners, following the 2006 Chicago Consensus Statement [4]. Because people with VSCs have bodies that are considered abnormal, they are subjected to medically unnecessary practices that may result in serious health issues (such as decreased sensitivity, incontinence, stigma, and serious mental health problems [4,5,6,7].

Claims that the rates of non-vital medical interventions on children have changed have been made since the 1980s [8,9] but have not been substantiated by the clinical data [10,11]. There is no consensus on the indications, timings, procedures, or evaluation of the outcomes of genital surgery [11]. Non-surgical care pathways for children receive little attention in practice or the literature. The scarce research on non-surgical care pathways for children is focused on medicalised approaches and often sidelines the role of psychologists.

The Chicago Consensus Statement introduced new guidelines for the care of children with VSCs and their families [4,5]. This statement has been internationally influential and remains a key reference point for care protocols and practice. It advocates for a multidisciplinary approach to care, involving a multidisciplinary team (MDT) of specialists such as endocrinologists, urologists, surgeons, psychologists, geneticists, and social workers. The statement emphasises the importance of psychosocial support from diagnosis through various stages of care, to help families make informed decisions and manage the psychological impact of a VSC diagnosis. It also advises caution with early non-vital surgeries on children, recommending that such surgeries be delayed until the individual can participate in decision-making. Open and ongoing communication with families is also stressed to ensure they are well-informed and supported [4,12].

In 2016, the consensus statement was updated, including greater recognition of the decision-making capabilities of minors. This was a shift towards supporting children’s rights, framing shared decision-making as central to patient-centred care. The update reinforced the idea of collaboration among the team members and patient-centred care to enable patients and their parents to make fully informed decisions, by drawing on the respect for participatory rights of children [13].

The consensus statement and its updates have been criticised for not addressing medical practices with adverse consequences for intersex people [3,5,14]. The continuation of unnecessary medical interventions on minors too young to give informed consent is recognised as a violation of human rights by human rights bodies, including the United Nations [6,7,15]. Human rights bodies have called for restrictions on medically unnecessary surgeries, implementation of anti-discrimination laws, the rights to private life and physical integrity, and respect for bodily autonomy [7,16]. Countries such as Malta, Portugal, Iceland, Germany, Greece, and Spain have legally prohibited medically unnecessary surgical interventions, but not all of them have defined legal consequences if the prohibitions are breached [17,18]. The legal restrictions on medical practices aim to support the implementation of improved medical care, including the provision of psychosocial support [19,20]. The usual MDT primarily includes clinical roles. This medicalised approach is problematic, as it is often provided at the expense of psychosocial support, which should be integral rather than optional [21].

Although there are concerns about the consensus statement and its update, the recommendations regarding psychosocial care may be useful, especially for supporting parents making decisions about their child’s care. Care provided should include comprehensive information about tests, treatment options, and potential side effects. It is important to compare the work of different MDT members, including healthcare professionals and psychologists, in relation to children’s and parent’s needs and the implementation of surgical and non-surgical care pathways. However, there is insufficient knowledge of best practice, psychosocial support (method, setting, timing), and healthcare professional’s opinions regarding psychosocial support [22,23].

This paper aims to address the gap in knowledge about psychosocial care and MDTs, contributing to discussions about healthcare for children with VSCs of all ages and their families, within a wider social context. To the authors’ knowledge, this is the first European-wide survey on this issue. The study is innovative in including peer supporters as well as healthcare professionals and psychosocial professionals, and in addressing obstacles to the provision of psychosocial and peer support. Furthermore, the study aims to compare the opinions on psychosocial support between healthcare professionals and peer support group members. Peer support is beneficial for people with VSCs, particularly minors with VSCs, as it provides emotional and social assistance, helps reduce feelings of isolation, and supports personal growth through the sharing of experiences. For parents and caregivers, peer support facilitates informed decision-making, offers emotional support during distressing times, and aids in navigating the healthcare system [24].

Psychosocial support addresses the emotional, psychological, spiritual, and social aspects of patient/service users and their families [25]. It is provided by mental health professionals, most often clinical psychologist and/or psychiatrists, social workers, and peer supporters. The provision of psychosocial support is seen as part of a MDT [4,26,27]. As indicated above, a team should include (paediatric) endocrinologists, urologists, surgeons, psychiatrists/psychologists, gynaecologists, geneticists, and neonatologists, and if available, social workers, nurses, and medical ethicists [4]. A MDT should educate other healthcare professionals involved in the treatment of people with VSCs, communicate with family members under supervision of a healthcare professional, and develop a plan for clinical management [4,26,28].

Psychosocial support is considered to be one of the most important aspects of care provided for minors with VSCs and their families [27], as it improves parental involvement in the care for children [29]. In addition, psychosocial support is considered fundamental for establishing a setting in which families can make well-informed decisions, given that families go through a spectrum of emotions when a VSC is diagnosed [30]. The importance of psychosocial support is also substantiated by research on women with Congenital Adrenal Hyperplasia (CAH) that showed how suitable psychosocial support impacts body image and general quality of life [31]. It should be noted that the adequacy of knowledge and skills amongst psychologists can be an issue, with reports of potentially harmful practice amongst psychologists in this field (for example, where a psychologist is complicit in enforcing a child’s compliance with neovaginal dilation [32]) but this topic is not the focus of this paper.

Despite the importance of psychosocial care for parents and their children with VSCs, psychosocial provision remains a scarce resource [27]. A multidisciplinary approach that includes psychosocial support has often not been adopted and adequately implemented. Existing research shows that parents perceived psychosocial support as important, but only half of them received it [33]. Some studies also indicate that the provision of psychosocial support is inadequate in terms of available resources and quality [22,23,33]. One issue is that psychosocial support is still based on a medicalised approach, with the parameters of the involvement of psychosocial support workers defined and constrained by healthcare professionals who have a leading role of organising, guiding, and managing consultations on VSCs [21,34]. Overall, the development and implementation of appropriate healthcare, including psychosocial care, is important for people with VSCs [35].

In addition to emphasising the need for psychosocial care, the consensus statement update stressed the importance of peer support groups for people with VSCs and their families to help reduce feelings of isolation and stigma and provide a safe space for discussions [26]. Peer support is used in a range of healthcare settings; it includes social and emotional assistance delivered with expertise by someone with personal experience [36]. This support is mutually agreed upon and delivered by individuals who self-identify as having, or having had, mental health or other social, psychological, and medical challenges. The aims are to facilitate self-determined personal change, and to support information sharing and the navigation of the healthcare system amongst service users facing similar challenges. Peer support proves beneficial in assisting parents navigate both the initial days and the enduring challenges of raising a child with VSCs [30], but there is a lack of evidence that it is provided when treatment options are discussed [37]. There are many different categories of peer support: self-help groups, online support groups, services provided by peers, services run or operated by peers, partnerships involving peers, and employment opportunities for peers [36].

To summarise, behavioural interventions—notably psychosocial support and peer support—can be seen to play a key role in supporting families and patients/service users with VSCs. As noted above, the continued practice of unnecessary medical interventions on minors too young to give informed consent is condemned by human rights bodies. Whilst healthcare professionals may claim that non-vital interventions on minors have ceased, this is disputed by some healthcare professionals and intersex activists [5,14,38]. This places the issue of psychosocial care and peer support in a highly significant position, given the role that these may have in fully informing parents and supporting them to make the right choices for their children in countries where there are no prohibitions on non-vital and potentially harmful interventions on VSC minor’s bodies.

## 2. Materials and Methods

This survey-based study was conducted between October 2022 and January 2023. Purposive selection procedures were used to select informants in the field of medical care for people with VSCs based in in Europe. The respondents were recruited via email by the team members using purposive sampling of DSD clinics and peer support groups, and information was circulated in the networks such as SDM registries and PSI I International. SDM Registries is a platform for sharing knowledge and experiences on rare conditions affecting sex development and maturation, enabling experts to collaborate and improve clinical practice, research, and understanding. PSI-I—Psychosocial Studies Intersex* International is a network that supports the mental well-being of people with variations in sex characteristics. We directly contacted over 65 VSC/DSD-related clinics and 70 peer support groups across Europe. The study targeted clinicians in countries as diverse as Denmark, Iceland, The Netherlands, the UK, Slovakia, Moldova, Germany, Italy, and Spain, as well as specialist medical networks and individual healthcare practitioners and psychosocial practitioners. The inclusion criteria for respondents were as follows:

- Healthcare professionals working in a European MDT providing care for children with VSCs and their parents;
- Members of peer support groups involved in any way in collaboration with a MDT and/or on issues concerning healthcare provision to minors with VSCS.

Exclusion criteria concerned age (no under 18 s, for ethical reasons); health and psychosocial care professionals not working directly with families who have a child with VSCs or with children/young people with VSCs; and peer support organisations that might be relevant (for example NGOs working on broad spectrum LGBTQI+ issues) but that did not work on issues concerning healthcare and MDTs for minors with VSCs. Questions about the ethnicity of survey respondents, or other demographic variables such as location, were not included because of concerns about maintaining confidentiality. Ethics approval was obtained in February 2022 by CEBES Review Board, the Ethics Committee of the Institute of Bioethics and the History of Medicine, University of Zurich.

### 2.1. Measures

Healthcare and psychosocial care professionals working with families with a child who has VSCs were asked about whether psychosocial care was offered at their practice, and if so, the type and timing of it. Peer support providers were asked about their knowledge of psychosocial care provision within medical institutions. Psychosocial support was defined in the survey as referring to the actions that address the psychological and social needs of individuals, families, and communities. Respondents were questioned about the provision of this support (with the answer options *current practice, aspired practice* or *not needed*). *Aspired practice* refers to practice that they would like to have in place. Respondents were asked about their views on whether there are substantial benefits in providing psychosocial support (*yes/no*). They were then asked about collaboration with peer support organisations (defined in the survey as in the Introduction to the current study, i.e., to be *when people use their own experiences to help each other* (multiple answer options) and if they thought that peer support could facilitate the decision-making of parents/legal guardians (*yes/no*). The survey also contained questions about the ways peer support could facilitate the decision-making of parents/legal guardians; the influences of different aspects on the decision-making process (such as parental age, education, and health status); and financial and logistical accessibility to quality healthcare (with the answer options *not at all influential*, *slightly influential*, *somewhat influential*, *very influential* or *extremely influential*). The respondents were then asked to indicate the frequency of barriers to access of healthcare (by means of a 5-point Likert item), to assess statements related to barriers for minors and their parents/legal guardians (with the answer options *agree* and *disagree*), and to provide answers about psychosocial and cultural issues that affect the provision of healthcare services. They were also asked to rate the extent of a range of different challenges faced by healthcare professionals (with the answer options *strongly disagree*, *disagree*, *neither agree nor disagree*, *agree* and *strongly agree*). Finally, respondents were asked whether or not they agreed with a set of general statements relating to the treatment of intersex persons (with answer options *agree* or *disagree*).

### 2.2. Statistical Analysis

The analysis comprised the following: (i) a characterisation of the sample using tabulated descriptive statistics and figures and (ii) a table summarising the results of simple inferential procedures. The sample was summarised descriptively, using tabulated data and cross-tabulations to summarise responses to categorical items. Key variables of interest, including *challenges faced by healthcare professionals* (elicited using 5-point Likert-style items); *methods of collaboration with healthcare professionals*; and *methods of collaboration with peer-support providers* (elicited as yes/no binary items) were represented graphically on an individual basis. The variable *challenges faced by healthcare professionals* was reported in terms of the proportion of respondents who reported either *agree* or *strongly agree* to a particular item. The relationship between the *type of psycho-social support* and *level of practice* (with options *current practice*, *aspired practice* or *not needed*); and between the *point of implementation of psychosocial support* and the *level of practice* was also represented graphically. The association between the *point of implementation of psychosocial support* and the *level of practice* was also tested using chi-squared testing (without a priori hypotheses). No corrections for multiple comparisons were made but may be applied informally. All analyses were based on valid responses only, i.e., a response that is not missing and that is selected from a list of permissible items. The statistical software used in this study was SPSS (Version 28). 

## 3. Results

Completed surveys were received from 301 respondents. The demographic and clinical variables collected included gender, age group, region of origin, best description of position (i.e., the broad categorisation of respondents as providers of either medical, psychosocial/social or peer support when in contact with minors with VSC/DSD and/or their parents or carers), best description of role (a more specific categorisation for example endocrinologist, urologist etc.), health facility and existence of a VSC/DSD MDT in their own institution. Many items were omitted by some respondents. For the purposes of this analysis, best description of position was selected as variable for the inferential analysis. A total of 222 respondents indicated their position: 65 (29.3% of those providing a valid response) as providers of medical care, 31 (14.0%) as providers of psychological/social services, 84 (37.8%) as provider of peer support, and 42 (18.9%) as other positions. In total, 121 respondents did not state their position. The sample is summarised descriptively in Table 1.

**Figure 1:**
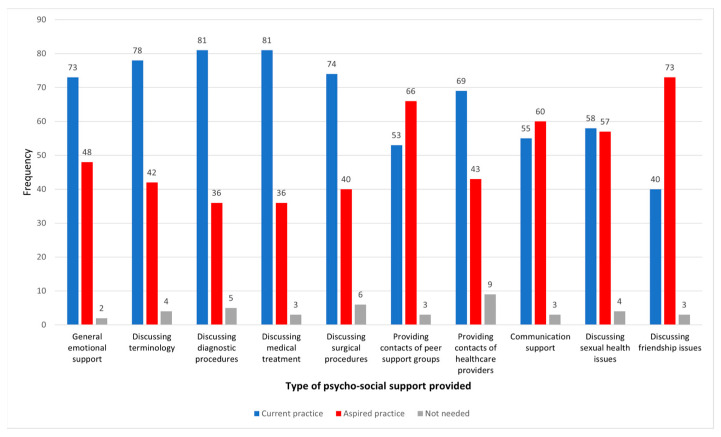
Type of psychosocial support.

Between 116 and 124 respondents gave valid responses to the item eliciting the type of psychosocial support provided to families of minors with VSCs (Figure 1). A very low number of respondents considered psychosocial support to be unnecessary. The most common features of current practice included discussing diagnostic procedures and medical treatment (81 respondents: 66.4%), with similar frequencies and proportions of general emotional support, discussing surgical procedures, and discussing terminology. Discussing friendship issues was an aspect of support that was aspired to substantially more often than it was actually provided (40 respondents: 34.5%).

**Figure 2:**
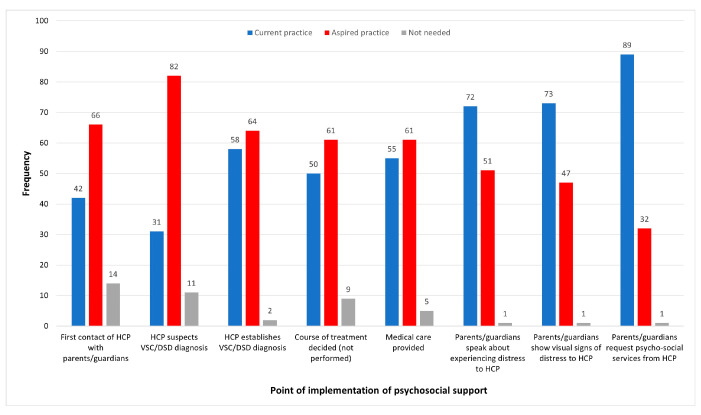
Point of implementation of psychosocial support.

Survey respondents were asked about the point of implementation of psychosocial support (Figure 2). Between 120 and 124 respondents gave valid responses to each of the items. The option *not needed* was selected by only a very small number of respondents in each case. Current practice appears to be skewed towards implementing psychosocial support primarily on request by parents/guardians (89 respondents: 73.0%), when parents/guardians speak about experiencing distress (72 respondents: 58.1%) or show visible signs of distress to healthcare practitioners (73 respondents: 60.3%). The most notable example of practice, which was aspired to, but not currently achieved, was the implementation of psychosocial support when a healthcare practitioner suspects a VSC/DSD diagnosis, aspired to by 82 respondents (66.1%), but current practice for only 31 (25.0%).

The results of the inferential analysis on the relationship between *best description of position* and *anticipated point of implementation of psychosocial support* are summarised in Table 2, with percentages based on distribution of responses of each type of provider within each time point. The implementation of psychosocial support was reported to be current practice at an average of 3.64 time points (from the list of time points in Figure 2); to be aspired practice at an average of 3.62 time points; and to be not needed at an average of 0.42 time points. Healthcare professionals and psychological/social services providers reported that the implementation of psychosocial support at all timepoints under consideration was current practice more commonly than any other response. The differences in distribution of responses across these two types of providers did not appear to be substantive. However, peer support providers reported that implementation of psychosocial support at all timepoints under consideration was aspired practice more commonly than any other response; this option amounted to over 70% of all responses associated with all timepoints, with one exception. Significant associations between best description of provision and status of practice were revealed at the 5% significance level for all tested anticipated points of implementation (*p* < 0.001 in all cases). Due to the low numbers of respondents who responded *not needed* to any of the tested items, this response was excluded from the inferential testing procedure. It may be inferred that the significant findings arose from differences in responses between providers of either medical care or psychological and social services on the one hand, and providers of peer support on the other hand.

**Figure 3:**
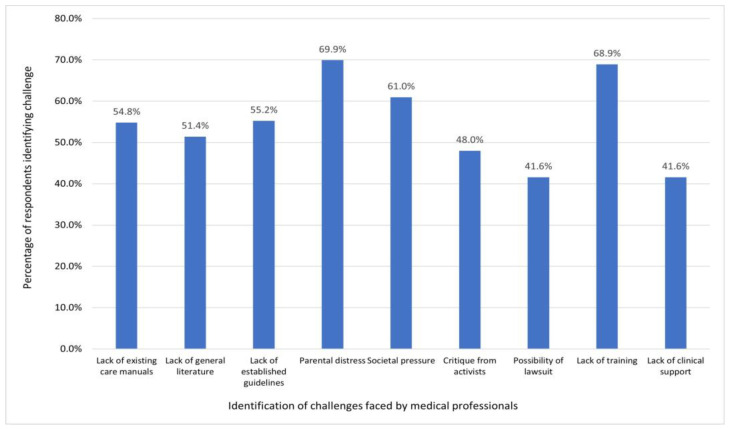
Identification of challenges faced by healthcare professionals.

Another question focused on the extent of challenges faced by healthcare professionals in providing adequate healthcare for minors with VSCs and their parents/legal guardians (Figure 3). A total of 110 valid responses were received and are summarised in Figure 3. Parental distress and lack of training appeared to be the most difficult challenges faced by healthcare professionals.

**Figure 4:**
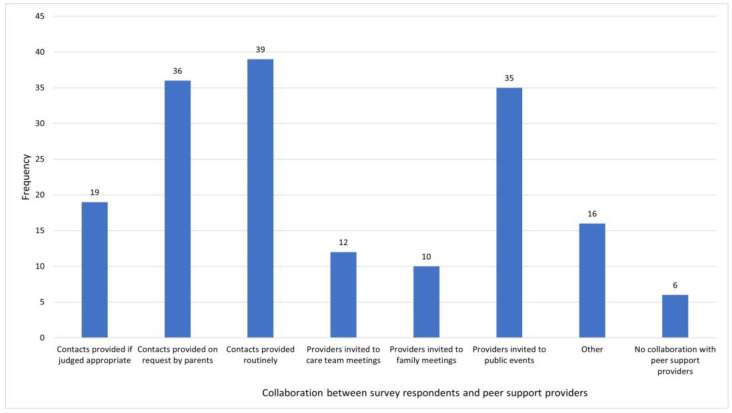
Methods of collaboration with peer support providers.

The survey also explored the relationship between healthcare and peer support provision. Providers of healthcare and psychosocial services were asked how they collaborate with peer providers when caring for minors with VSCs and their parents. In total, 72 respondents provided 1 or more valid responses, summarised in Figure 4. Routine contact, contact provided on request and invitations to providers to attend public events were the most commonly reported methods of collaboration, all reported by about 50% of respondents. Six respondents (8.3% of those providing a valid response) stated that they do not collaborate with peer support providers. Whilst some healthcare and psychosocial care providers collaborate with peer supporters to a substantial extent, more extensive collaboration, such as involvement in team meetings, was less common.

**Figure 5:**
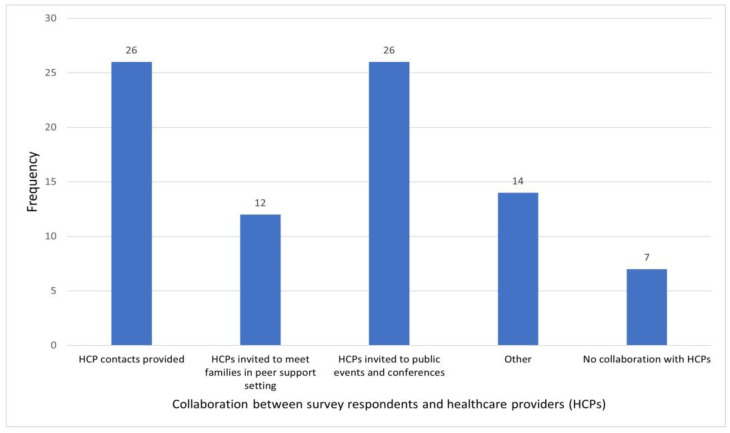
Methods of collaboration with healthcare providers.

Peer providers were asked in which way they collaborate with healthcare professionals when caring for minors with VSCs and the 48 respondents provided 1 or more valid responses, as summarised in Figure 5. More than half of respondents reported providing contact details of healthcare professionals, on request or routinely, or inviting them to public events (such as lectures and roundtable discussions) and conferences. Seven respondents (14.3% of those providing a valid response) stated that they do not collaborate with healthcare professionals (Figure 5).

## 4. Discussion 

This original exploratory trans-European survey provided data about the views of healthcare and psychosocial providers of care to minors of all ages and their families. These respondents were primarily based at public hospitals and in specialised VSC/DSD care teams. A range of roles were represented with a predominance of endocrinologists, psychologists, and paediatricians. The study is innovative in including peer support organisations, providing insight into the differences of opinion between groups of respondents. The peer support organisations included patient and parent support groups, and activist organisations.

This paper reported on findings in the following areas: type of psychosocial support; point at which psychosocial support was implemented; associations between professional position and points of implementation of psychosocial support; identification of challenges faced by healthcare professionals; and methods of collaboration between peer support providers and healthcare professionals.

Overall, the results indicated that the majority of respondents viewed psychosocial support as important but that there is insufficient provision. The data revealed a gap between aspirations for the provision of psychosocial support and the support that is currently offered. Given the findings from previous studies that indicate that lack of adequate psychosocial and peer support can have serious consequences, this is of concern. For example, one study [37] showed that parents of children with VSCs experienced decisional regret regarding non-vital medical interventions because they did not receive adequate peer and psychosocial support, particularly prior to any medical interventions taking place. The survey findings also indicated that the provision of peer support lags behind medical care and psychosocial support despite the recognition that peer support is important for parents/guardians and children with VSCs in the consensus statement update, as noted above.

Another issue raised in the survey findings is that that psychosocial and peer support regarding friendship issues is much more likely to be aspired to in practice than other types of support. What seems to be missing is psychosocial support that addresses issues surrounds navigating day to day upbringing, early years care, peer relations, education, dating, and extended family relations. These issues could be addressed by involving social workers in the collaboration process. The findings about support regarding friendships were raised in a previous study which indicated that children disclosed information about their VSCs to friends, whom they considered to be the second most important people in their lives, after their mother [39].

This study showed that healthcare and psychosocial professionals believe that alignment between desired and actual psychosocial support is fairly close when parents/legal guardians are distressed or actively seeking support. However, this alignment tends to be less robust when healthcare professionals first encounter parents/legal guardians, and when VSCs are suspected or diagnosed. This can be highly problematic as parents/legal guardians need excellent psychosocial support when making decisions about their child’s care pathway or if VSCs are indicated during prenatal examinations. It is also important to note that psychosocial support is a continuous requirement, as parents/legal guardians and children have concerns about the upbringing and relationships long after the diagnosis, as indicated in the literature [30]. Moreover, continuous psychosocial support is considered integral to multidisciplinary care, and previous findings highlight that multidisciplinary care is associated with overall satisfaction with care [40]. The majority of psychosocial support currently provided appears to be in responses to events that have taken place after surgical interventions. This finding points to problematic practice, especially given the human rights imperatives to preserve the bodily integrity of minors with VSCs (see above). Overall, the timing of psychosocial care, which should be provided as soon as a diagnosis is suspected or made, is crucial.

Inferential analysis of the survey data revealed a significant difference between the provision of psychosocial support as perceived by healthcare professionals and psychosocial providers, and provision as perceived by peer support group members. Healthcare professional and psychosocial providers stated that in their opinion, psychosocial support is provided multiple times, whereas members of peer support groups did not experience this, and they wished for the provision of psychosocial care at multiple times. This finding is novel and adds knowledge about different attitudes and experience of psychosocial care for minors with VSCs and their families across Europe. Differences in perception could be explained by the structure of care for minors with VSCs that still centres around biomedical assessment and treatment, while psychosocial support is sidelined [21]. It could also be related to the physical remoteness of available peer support services [41].

Challenges to practice were identified by healthcare professionals contributing to the survey, with several specific challenges identified by over 50% of the sample including parental distress, lack of training, societal pressure, lack of established guidelines, lack of existing care manuals and a lack of general literature. This is consistent with the previous findings on collaboration between MDT team members, minors with VSCs and parents [42]. The findings complement the literature regarding challenges in this field as exacerbated by disparities in funding, with medical specialties such as psychiatry, paediatrics, pathology, and neurology receiving more financial support compared to family medicine and gynaecology [43,44]. The survey also provided new data showing that over 40% of healthcare professionals viewed critique from activists and the possibility of lawsuits as challenges. This is important, because psychosocial care and peer support can help informed decision-making as means to not only support better care for minors with VSCs, but also to avoid critique of healthcare professionals and possible litigation. 

The survey showed that healthcare professionals and peer support groups stated that they most often collaborate on routine basis (with contacts most often established by healthcare professionals). They also collaborate with peer supporters when parents/legal guardians request that kind of support, and when peer support providers are invited to public events. This is in accordance with previous findings that highlighted the need for familial support by non-healthcare professionals [39,41]. Healthcare and psychosocial care respondents reported that peer support collaborations consisted mainly of healthcare professionals providing contacts to peer support groups, and other types of collaboration featured substantially less. Long-term collaboration seems to be much less developed, which is concerning, given the ongoing needs that people with VSCs and their families have. These findings corroborate and add to previous research on opinions regarding the organisation of psychosocial support in which the healthcare professionals and members of peer support agreed that the psychosocial support should be organised and continuously provided [41], and by studies showing positive effects on parents and people with VSCs [31,37,45].

This study has some limitations. The use of purposive sampling and the low response rates to certain survey items both limit the extent of the inferences possible from the data. As noted above, some roles are overrepresented (notably endocrinologists) and the representation across different countries was varied, as would be expected from an exploratory study. The findings regarding an association between type of provision and status of practice can be seen to be grounded in the difference between peer support practice on the one hand, and medical care/psychosocial support on the other hand. However, with no a priori hypotheses, the uncontrolled significance testing is viewed as exploratory and complements the findings of the descriptive analyses.

## 5. Conclusions

Psychosocial support and peer support are rated as important by healthcare professionals and members of peer support groups, but there are deficits in provision, almost 20 years after the consensus statement was introduced. The survey findings indicate that there is a need for more psychosocial support when a VSC is suspected or diagnosed, to help parents/legal guardians in decision-making about their child’s care. This is particularly important, given issues of potential parental/legal guardian regret, and the worries that healthcare practitioners have about potential litigation. There were differences of opinion amongst respondent groups, with healthcare professionals and psychosocial care professionals reporting higher levels of psychosocial support provision than peer support organisations. The survey also showed that collaboration with between health care professionals and peer support organisations is quite common, but that this is often restricted to the provision of contacts, without peer supporters being involved in more substantial ways. Although this study did not address resource constraints regarding psychosocial care and peer support specifically, the analysis of data and the adjacent literature indicates a need for more funding for these aspects of care. Overall, the deficits in psychosocial care imply a need for policy makers and practitioners to implement more robust psychosocial care and peer support for children with VSCs and their families across Europe and their families.

## Figures and Tables

**Table 1:** Descriptive summary of sample.

Variable	Frequency (Valid %)
Gender: not specified as intersex (*n* = 67)	
Female	40 (59.7%)
Female/non-binary	5 (7.5%)
Male	14 (20.9%)
Male/non-binary	1 (1.5%)
Non-binary	7 (10.4%)
Gender: specified as intersex (*n* = 16)	
Female	5 (31.3%)
Male	3 (18.8%)
Non-binary	3 (18.8%)
No gender designation	5 (31.3%)
Age group (*n* = 108)	
26–40	37 (34.3%)
41–60	52 (48.1%)
61–70	17 (15.7%)
>70	2 (1.9%)
Region of origin (*n* = 107)	
Asia	4 (3.7%)
Australia/New Zealand	4 (3.7%)
Europe	96 (89.7%)
Europe/Africa	1 (0.9%)
United States	2 (1.9%)
Best description of position (*n* = 222)	
Provider of medical care	65 (29.3%)
Provider of psychological/social services	31 (14.0%)
Provider of peer support	84 (37.8%)
Other	42 (18.9%)
Best description of role (*n* = 96)	
Geneticist	3 (3.1%)
Gynaecologist	3 (3.1%)
Endocrinologist	23 (24.0%)
Ethicist	1 (1.0%)
Surgeon	6 (6.3%)
Paediatrician	10 (10.4%)
Psychologist	14 (14.6%)
Psychiatrist	4 (4.2%)
Social worker	3 (3.1%)
Urologist	7 (7.3%)
Other ^1^	22 (22.9%)
Health facility (*n* = 95)	
Public university hospital	61 (64.2%)
Public non-university hospital	17 (17.9%)
Private institution/hospital	6 (6.3%)
Other	11 (11.6%)
Specialised/multidisciplinary VSC/DSD care team in institution (*n* = 94)	
Yes	72 (76.6%)
No	18 (19.1%)
Not sure	4 (4.3%)

^1^ Other roles specified (*n* = 3 or fewer in all cases) included counsellor, dietitian, electrologist, family therapist, nurse, researcher (non-specific), speech and language therapist, special/rehabilitative pedagogist, support worker/personal assistant.

**Table 2:** Associations between position and points of implementation of psychosocial support.

Anticipated Point of Implementation of Psychosocial Support	Status	Best Description of Provision			Test Result
		Medical Care	Psychological/Social Services	Peer Support	
First contact of healthcare professional with family	Current practice	30 (52.6%)	9 (47.4%)	3 (6.4%)	χ^2^_(2)_ = 26.1, *p* < 0.001
	Aspired practice	21 (36.8%)	9 (47.4%)	37 (78.7%)	
	Not needed	6 (10.5%)	1 (5.3%)	7 (14.9%)	
Establishment of VSC/DSD diagnosis by healthcare professional	Current practice	40 (71.4%)	12 (63.2%)	6 (12.2%)	χ^2^_(2)_ = 39.5, *p* < 0.001
	Aspired practice	15 (26.8%)	7 (36.8%)	42 (85.7%)	
	Not needed	1 (1.8%)	0 (0.0%)	1 (2.0%)	
Course of treatment decided but not performed	Current practice	34 (61.8%)	10 (55.6%)	6 (12.5%)	χ^2^_(2)_ = 29.9, *p* < 0.001
	Aspired practice	17 (30.9%)	6 (33.3%)	39 (81.3%)	
	Not needed	4 (7.3%)	2 (11.1%)	1 (6.3%)	
Achievement of proper medical care	Current practice	37 (68.5%)	11 (57.9%)	7 (14.6%)	χ^2^_(2)_ = 31.1, *p* < 0.001
	Aspired practice	16 (29.6%)	7 (36.8%)	39 (81.3%)	
	Not needed	1 (1.9%)	1 (5.3%)	2 (4.2%)	
Speaking about experiencing distress to healthcare professional	Current practice	46 (80.7%)	16 (84.2%)	11 (22.4%)	χ^2^_(2)_ = 41.9, *p* < 0.001
	Aspired practice	11 (19.3%)	3 (15.8%)	37 (75.5%)	
	Not needed	0 (0.0%)	0 (0.0%)	1 (2.0%)	
Parents/guardians showing visual signs of distress to healthcare professional	Current practice	45 (80.4%)	16 (84.2%)	13 (27.1%)	χ^2^_(2)_ = 35.0, *p* < 0.001
	Aspired practice	11 (19.6%)	3 (15.8%)	34 (70.8%)	
	Not needed	0 (0.0%)	0 (0.0%)	1 (2.1%)	
Parents request psychosocial services from healthcare professional	Current practice	49 (87.5%)	19 (100.0%)	22 (44.9%)	χ^2^_(2)_ = 31.1, *p* < 0.001
	Aspired practice	7 (12.5%)	0 (0.0%)	26 (53.1%)	
	Not needed	0 (0.0%)	0 (0.0%)	1 (2.0%)	

## Data Availability

The survey, and the data that support the findings of this study, are available on request from the corresponding author, S.M. The data are not publicly available as they contain information that could compromise the privacy of research respondents.

## Abbreviations

DSD	Disorders/differences of sex development
HCP	Health care professional
ICD	International Classification of Diseases
MDT	Multidisciplinary team
VSC	Variations of sex characteristics
